# Eyelid Retraction in Isolated Unilateral Congenital Blepharoptosis

**DOI:** 10.3389/fneur.2017.00190

**Published:** 2017-05-05

**Authors:** Michael S. Salman, Ian H. Clark

**Affiliations:** ^1^Section of Pediatric Neurology, Children’s Hospital, University of Manitoba, Winnipeg, MB, Canada; ^2^Department of Pediatrics and Child Health, Max Rady College of Medicine, Rady Faculty of Health Sciences, University of Manitoba, Winnipeg, MB, Canada; ^3^Section of Pediatric Ophthalmology, Children’s Hospital, University of Manitoba, Winnipeg, MB, Canada; ^4^Department of Ophthalmology, Max Rady College of Medicine, Rady Faculty of Health Sciences, University of Manitoba, Winnipeg, MB, Canada

**Keywords:** isolated, congenital, blepharoptosis, levator palpebrae superioris, lid retraction in downgaze, static eyelid

## Abstract

Isolated unilateral congenital ptosis is encountered relatively infrequently in clinical practice. It typically consists of a unilateral droopy eyelid, weak levator palpebrae superioris muscle function, lid lag, and an absent upper lid crease with no other abnormalities on examination. We present a four-and-a-half-year-old girl with isolated and mild unilateral congenital ptosis who unexpectedly demonstrated a static upper eyelid on downgaze in conjunction with a well-formed upper lid skin crease. We attribute this uncommon sign in congenital ptosis to stiffness and presumed fibrosis of the levator muscle. Examining the function of the eyelids in all directions of gaze is important in patients with abnormalities of lid position, since additional useful information can be gleaned about the status of the levator muscle including, aberrant regeneration or fibrosis.

## Introduction

Upper eyelid blepharoptosis, commonly referred to as ptosis, is seen relatively commonly by ophthalmologists and neurologists. It is defined as an inferior malposition of the upper eyelid margin with respect to the superior corneo-scleral limbus in the absence of another cause, such as a hypotropia or enophthalmos. Isolated congenital ptosis, on the other hand, is encountered less frequently. It is present at birth but often goes unnoticed in the first few months of life (1). There are other causes of ptosis in a newborn, including a third cranial nerve palsy and the Marcus Gunn jaw-winking syndrome, but these are distinct from isolated congenital ptosis, which is the subject of this paper.

Isolated congenital ptosis typically consists of a unilateral ptosis with weak levator palpebrae superioris muscle function (seen when looking up with the frontalis muscle neutralized by exerting pressure on the ipsilateral eyebrow), lid lag on downgaze, and often an absent upper lid crease (2). The rest of the neuro-ophthalmological and neurological examination is normal. It should be mentioned that ptosis of the lower eyelid is a recognized entity; however, this paper will be referring only to ptosis of the upper eyelid.

Isolated congenital ptosis is usually sporadic but may be familial with no well-defined pattern of inheritance. The cause of isolated congenital ptosis was blamed on a presumed myopathy of the levator muscle but this view has been challenged (3), and recent evidence suggests a developmental genetic abnormality in levator muscle innervation (4, 5). The histological changes are now thought to be neurogenic in origin, and the direct consequence of defective innervation. The affected muscle is usually dystrophic, with fibrous and fatty tissue replacing the normal muscle fibers, resulting in an abnormal muscle that contracts and relaxes poorly. Surgery to correct ptosis is undertaken in patients who are at risk of amblyopia, i.e., when ptosis is interfering with visual development or for cosmetic reasons. Surgery can only address the lid height (i.e., the palpebral aperture), the lid contour, and create a lid crease, if needed. It cannot restore normal contraction and relaxation of a dystrophic levator muscle.

We present a child with an apparently mild isolated unilateral congenital ptosis but whose upper eyelid appeared static on downgaze.

## Case Report

The patient was first brought to the attention of pediatric ophthalmology at 10 months of age. She was referred by her family physician because her parents noticed that her right upper eyelid did not depress on downgaze and it failed to close completely during sleep. It had been noted for several months prior to the initial visit and was first seen soon after birth. The eyelids closed fully when she cried but less tightly on the right. The parents had no other concerns about her health or vision.

The pregnancy had been normal with the exception of pregnancy-induced hypertension at 35 weeks for which the mother was treated with labetalol. The mother did not smoke, use any prescription or recreational drugs, and did not drink alcohol. The patient was born at 37-week gestation following spontaneous vaginal delivery with vacuum assistance. Birth weight was 2.33 kg. There were no complications in the post-partum period. The parents were non-consanguineous and were both 33 years old. The father is of Mennonite origin and mother is Irish/Welsh. The family ocular history was significant only for a second cousin whose lid also did not close fully but this individual has not been examined by the authors and no more details are available. Our patient was otherwise well and was developing normally.

On initial examination, the patient was orthophoric and her visual acuity was central, steady, and maintained bilaterally. Pupils were equal and reactive to light and there was no afferent pupillary light defect. There was no limitation of her ocular motility. There was a 0.5 mm ptosis of the right upper eyelid in primary gaze. Her right upper lid crease was noted to be normally formed, and symmetrical with the left upper lid crease. Her fundi were normal and eyelid movements were full but with poor right levator function noted in upgaze, and no aberrant movements. There was no evidence of exposure keratopathy. Cycloplegic refraction was +2.25/+2.25 × 090 OD and +1.50/+0.25 × 090 OS. There were no signs of involvement of the facial nerves. The patient was prescribed glasses for the mild anisometropia. Blood work for T3, T4, and TSH was normal. Old photographs from early infancy showed right lagophthalmos measuring approximately 2 mm.

On follow up at age 14 months, the right upper lid was no longer ptotic, but now appeared to be retracted by 0.5–1 mm. There was lid lag of the right upper lid in downgaze with associated scleral show and little movement of the right upper lid with reflex blinking. Additionally, manual traction on the lashes of the right upper eyelid identified a restriction in the levator muscle. There was a suggestion of a right hypotropia and exotropia at times although the eyes appeared orthotropic in the primary position for near and distance targets. The remainder of the examination was unchanged. Her thyroid function tests were repeated and they remained normal. The patient was wearing her glasses, as prescribed, and there was no suggestion of amblyopia. Her parents opted to defer neuroimaging due to sedation concerns and the patient was referred to pediatric neurology for a second opinion.

She was seen by pediatric neurology at age 16 months. There were no neurocutaneous stigmata or dysmorphic features. Her visual behavior appeared normal. Extraocular movements were full and pupils were equal and reactive to light. The pupils remained equal in size in all directions of gaze. The right upper eyelid moved minimally on downgaze with almost no movement on upgaze. At this time, she was noted to have a smaller palpebral aperture on the right, suggestive of a mild ptosis, but it was not possible to measure due to poor cooperation. Corneal reflexes through direct and indirect corneal stimulation were intact but elicited incomplete (partial) blinks on the right. She was noted to blink spontaneously bilaterally but asymmetrically, with partial blinks on the right. She displayed normal facial movements. She could produce tears with crying and saliva was present in her mouth. Her tone, strength, coordination, and reflexes were normal. An MRI of the brain and orbits, with thin cuts of 0.8 mm, showed no abnormalities.

**Figure 1:**
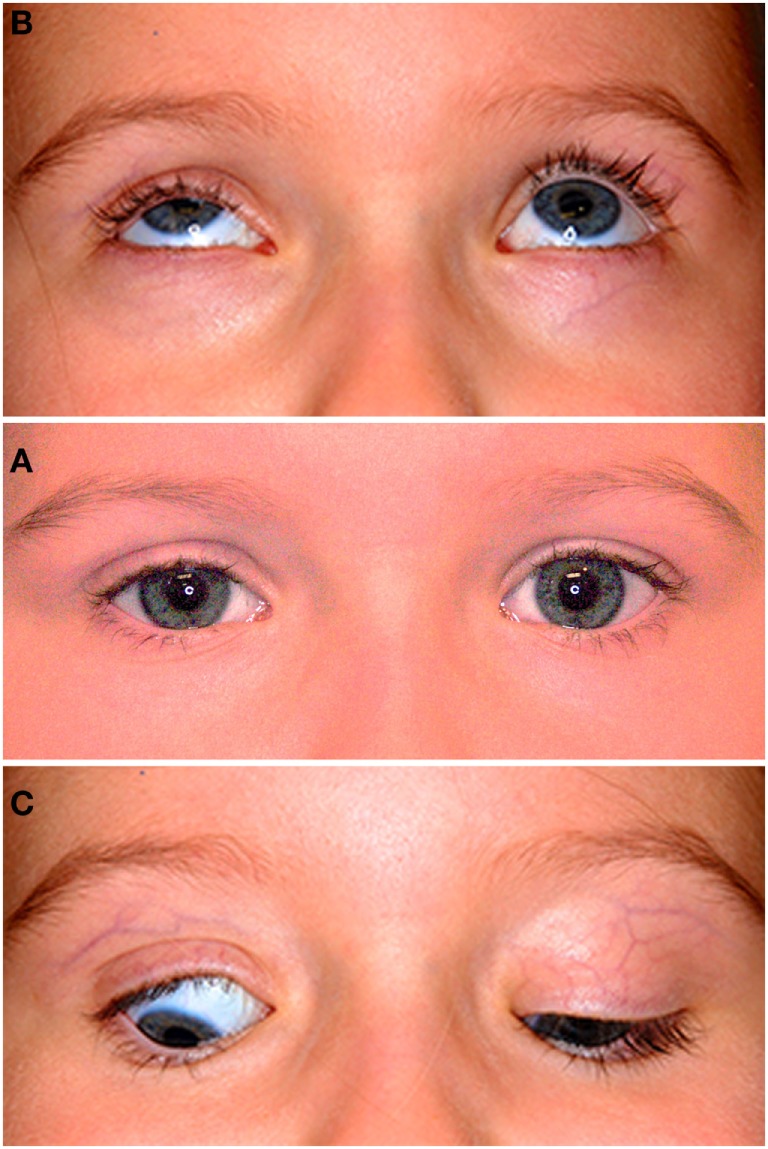
**The middle photograph (A) shows mild right ptosis as seen in the primary position at age 4-years and 6-months; note the well-formed skin crease on both sides, with the crease 1 mm higher on the right**. The upper photograph **(B)** shows poor levator function in upgaze on the right and normal elevation of both eyes. The lower photograph **(C)** shows restriction of the right upper lid in downgaze resulting in lid lag and scleral show; note the persistent lid crease on that side.

**Figure 2:**
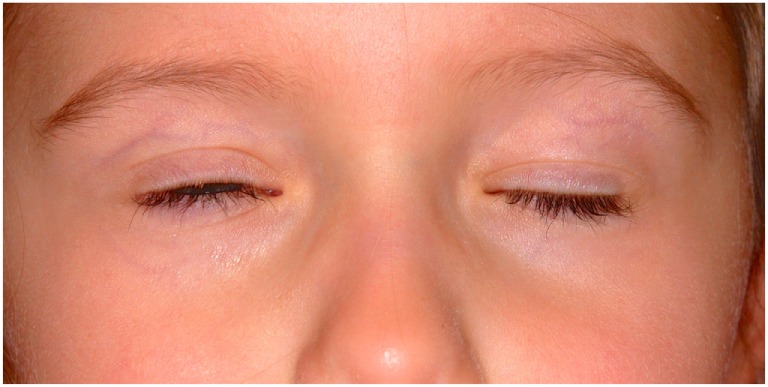
**The photograph shows lagophthalmos on the right at age 4 years and 6 months**.

The patient was followed over the next 3 years, and during this time, the right upper lid has become gradually more ptotic, by approximately 3 mm. Her lid crease remained well-formed and almost symmetrical, measuring 3 mm on the right and 2 mm on the left. Her right levator function measured 5 mm compared to 11 mm on the left. No synkinesis of the right levator was evident and the right upper lid was static on downgaze, with no relaxation of the levator resulting in scleral show (Figure 1) and lagophthalmos of approximately 1 mm (Figure 2). Her right eye started to show evidence of amblyopia at 33 months of age despite good cooperation with the use of her glasses. This responded well to patching of the left eye and her acuities were 6/9+ 2 OD and 6/7.5−1 OS at 54 months of age, with 80 arc seconds of stereopsis, orthophoria in all directions of gaze, and normal ocular motility. In the primary position, the mid-palpebral aperture measured 7 mm on the right and 10 mm of the left at the last clinic follow up.

## Discussion

Over the last two decades, congenital ptosis has been reclassified and is currently considered to be one of the congenital cranial dysinnervation disorders. The roles of several genes have been elucidated and found to have important or essential roles in the development of the brainstem, specific cranial nerve nuclei, or their axonal connections to their target, usually muscles (6). We refer the interested reader to several excellent reviews on the topic (6–8).

Ptosis in the first year of life may be caused by trauma (e.g., birth injury) or may have other etiologies that are myogenic (e.g., congenital myopathy or myasthenia gravis), syndromic, metabolic, mechanical (e.g., from a tumor), or neurogenic (e.g., congenital Horner syndrome or congenital oculomotor nerve palsy) (1).

In this paper, we present a child with isolated, unilateral, non-syndromic, congenital ptosis with no other ocular or neurological features. The upper eyelid did not cover the pupil, i.e., her ptosis was mild, to the extent that it was not apparent until after the first year of life. The right levator muscle was stiff (since the upper eyelid moved minimally on downgaze despite her mild ptosis) and fibrosed (evidenced by the palpable restriction when pulling on the upper lid). The effects of the resultant restriction were apparent soon after birth and well before her ptosis became apparent. There was almost no movement of the right eyelid on upgaze, consistent with weak levator function, and minimal movement of the upper lid on downgaze.

No evidence of synkinesis was seen on careful examination. We specifically looked for evidence of levator and inferior rectus synkinesis and found none. The right upper eyelid moved only minimally downwards on downgaze. On careful clinical examination aided by frame-by-frame analysis of a video recording of the patient’s eye movements, the right upper eyelid did not move upwards at any time when the child was looking down.

The corneal reflex response and blinks were asymmetrical, with partial response on the right due to stiffness of the levator. The action of orbicularis oculi, which was intact in our patient, overcame the levator stiffness during blinks and reflex blinking caused by corneal stimulation, albeit incompletely. During sleep, the mother reported either complete or incomplete closure (~70–80%) of the right eyelids i.e., she had lagophthalmos (9), which can be attributed to the stiffness of the right levator.

Our patient is unusual in that she has only mild ptosis, with poor levator function, yet a well-formed skin crease. She also demonstrates a distinctly stiff upper eyelid, which moved sluggishly and minimally on downgaze. We undertook a review of the medical literature on congenital ptosis and scrutinized photographs of children and adults with congenital ptosis in the primary position and downgaze, where available. It was very uncommon to see the sclera superior to the limbus of the effected eye in downgaze, i.e., lid retraction or static lid in downgaze, also referred to a “hang-up in downgaze” (10). Hang-up in downgaze is commonly seen after surgery for congenital ptosis. It has also been described in acquired ptosis associated with orbital malignancy or trauma (10), but it is an uncommon finding in unoperated congenital ptosis.

On the other hand, lid lag and a palpebral aperture that remains unchanged or increases on downgaze have been described in congenital ptosis (11, 12). In such patients, the ptotic eyelid usually partially covers the superior limbus in downgaze in contrast to our patient. We found different definitions of lid lag, with variations in the usage of the term. In one study, the authors defined lid lag as a dynamic phenomenon, seen during eye movement testing, consisting of a phase lag of the ptotic eyelid seen only during eye movement from upgaze to downgaze; the upper eyelid margin would be seen to catch up soon after the cessation of the eye movement (10). Other authors have labeled the aforementioned sign as the von Graefe’s sign (9), which they defined as retarded eyelid descent during a downgaze movement. In the same study, lid lag was defined as a static phenomenon, in which the upper eyelid position assumes a position higher than normal while the eyes are in downgaze (9). To avoid the confusion associated with the variations in the definition of these ocular signs, we simply describe our findings as follows: In our patient, the upper eyelid margin remained retracted after its initial slow and minimal downward movement on downgaze.

It is also notable that her skin crease was well-formed bilaterally despite her very poor levator function. A poorly formed skin crease is usually an indicator or poor levator function, but that was clearly not the case with our patient, whose levator function was poor, but her skin crease very prominent.

The appreciation of the fibrotic state of our patient’s levator has important management implications as it can be expected that any ptosis surgery will have a high probability of causing lagophthalmos due to the rigid nature of the muscle.

The etiology of the unilateral isolated congenital ptosis in our patient is unknown. There was no history of birth trauma, no evidence of dysmorphic features, of other ocular signs, or of a myopathic disorder. We, therefore, assume that it is neurogenic in origin, caused by abnormal innervation of the right levator palpebrae superioris muscle. Of interest, two genetic loci (*PTOS1 and PTOS2*) have been described in patients with congenital ptosis. Inheritance is autosomal dominant and X-linked dominant, respectively. The phenotype consists of unilateral or bilateral ptosis of variable degree in the former and severe bilateral ptosis, frontalis muscle overactivity, and chin-up head posture in the latter (13).

We suggest that examining the function of the eyelids in all directions of gaze is important in patients with ptosis, even when the ptosis is mild, since important information can be gleaned about the status of the levator muscle, namely aberrant regeneration or the presence and severity of fibrosis. Increasing stiffness of the levator may herald the onset of ptosis, so clinical follow-up is important.

## Ethics Statement

Mother gave verbal and written permission to publish manuscript and figures.

## Author Contributions

MS examined patient and wrote first draft. He edited subsequent versions of the manuscript. IC examined patient and contributed to the case report and, intellectually, to the discussion. He obtained both figures. Both approved the final version.

## Conflict of Interest Statement

The authors declare that the research was conducted in the absence of any commercial or financial relationships that could be construed as a potential conflict of interest.
